# Discovery of coniferaldehyde as an inhibitor of caseinolytic protease to combat *Staphylococcus aureus* infections

**DOI:** 10.1186/s10020-025-01306-2

**Published:** 2025-06-30

**Authors:** Shufang Li, Yan Zhang, Jianfeng Wang, Hongfa Lv, Hongxia Ma, Lingcong Kong, Yonglin Zhou, Jingmin Gu, Wei Li, Qiaoling Zhang

**Affiliations:** 1 Department of Gastrocolorectal Surgery, General Surgery Center, The First Hospital of Jilin University, Changchun, 130062 China; 2 State Key Laboratory for Diagnosis and Treatment of Severe Zoonotic Infectious Diseases, Key Laboratory for Zoonosis Research of the Ministry of Education, Institute of Zoonosis, and College of Veterinary Medicine, Jilin University, Changchun, 130062 China; 3 Hospital of Stomatology, Jilin University, Changchun, 130062 China; 4 College of Life Science, Jilin Agricultural University, Changchun, 130118 China; 5 Key Laboratory of Ministry of Education for Conservation and Utilization of Special Biological Resources in the Western China, and School of Life Sciences, Ningxia University, Yinchuan, 750021 China

**Keywords:** *Staphylococcus aureus*, Caseinolytic protease, Coniferaldehyde, Molecular simulations, Virulence

## Abstract

The rising incidence of methicillin-resistant *Staphylococcus aureus* (MRSA) poses a significant threat to global public health, highlighting the urgent need for novel therapies and treatments in clinical settings. Caseinolytic protease P (ClpP) serves as a key component of bacterial degradation systems, playing a crucial role in maintaining cellular homeostasis and contributing to pathogenicity. Targeting ClpP function inhibition has demonstrated potential in combating antibiotic resistance and offers a promising therapeutic strategy for treating *S. aureus* infections. In this study, coniferaldehyde (CA) was identified as a ClpP inhibitor through ClpP peptidase inhibition assay. CA reduced the hemolysis activity, protease hydrolysis and bacterial invasion ability via regulating the transcription of main virulence factors. Furthermore, CA treatment led to a decreased resistance of *S. aureus* to adverse stimuli, including heat, acidic pH, high osmotic environment, hydrogen peroxide and NaClO stress assays. Notably, CA enhanced the efficacy of the bactericidal antibiotic tigecycline against growing *S. aureus* in time-killing assays. Molecular simulations and mutagenesis analyses revealed that the amino acids M31 and G33 were critical for the interaction between CA and ClpP. Importantly, CA exhibited excellent protective efficacy against *S. aureus* pneumonia in murine infection models. Our findings confirm that CA is an effective ClpP inhibitor with potential as a therapeutic agent for *S. aureus* infections.

**Graphical Abstract:**

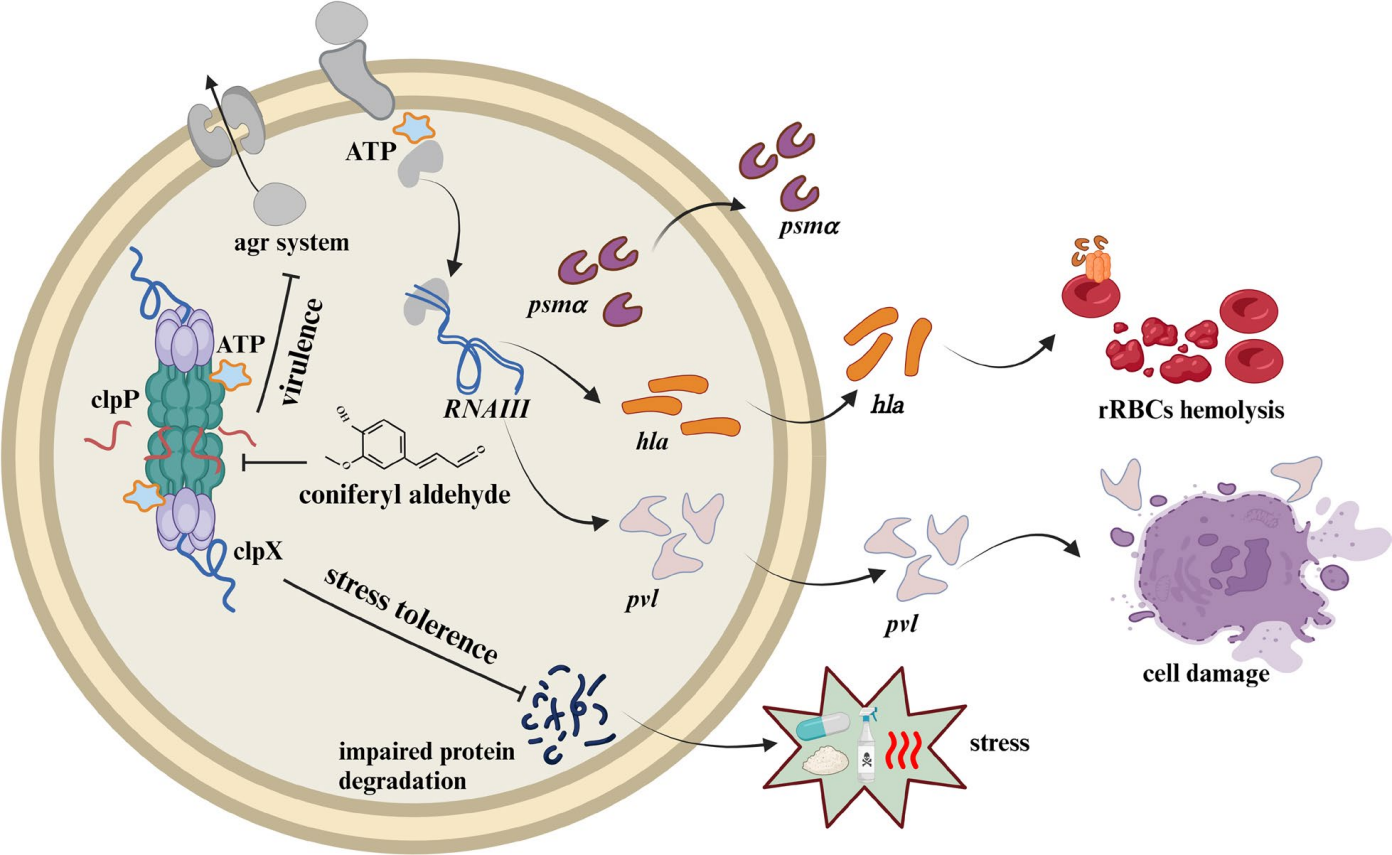

## Introduction

*Staphylococcus aureus* (*S. aureus*) poses a considerable public health risk due to its significant antibiotic resistance and ability to cause severe infections (Tong et al. 2015). Methicillin-resistant *S. aureus* (MRSA) is linked to a range of health problems, from minor skin infections to serious conditions, including pneumonia, bloodstream infections, and surgical site infections (Chambers and Deleo 2009). The rising incidence of antibiotic-resistant strains further complicates treatment, resulting in increased morbidity and mortality, particularly among vulnerable groups like the elderly and those with weakened immune systems (Vestergaard et al. 2019). The threat posed by MRSA is further exacerbated by antibiotics abuse in both healthcare settings and the community, with the limited number of effective treatment alternatives (Frieri et al. 2017). This creates a pressing need for novel therapeutic strategies to combat MRSA infections (Zhang et al. 2014; Kahan et al. 2021; Jiang et al. 2023).

ClpP, as a promising target for antibacterial and anticancer therapy, has attracted extensive research interest. Currently, ClpP modulators from different categories of chemical structures are being explored and developed for potential clinical applications (Brotz-Oesterhelt and Vorbach 2021). As a chymotrypsin-like serine protease, it has been thoroughly studied for its involvement in regulating virulence and stress responses in several bacterial species, such as *S. aureus* (Frees et al. 2003), *Listeria monocytogenes* (Gaillot et al. 2000), and *Streptococcus pneumoniae* (Kwon et al. 2004). ClpP is responsible for the degradation of proteins that have been mistranslated, misfolded, or aggregated in the bacterial cell as a result of e.g. heat stress or antibiotic interference with the ribosomal machinery (Aljghami et al. 2022). Knockout of the *clpP* gene in *S. aureus* weakened virulence in a murine skin abscess model, and a similar action was obtained after suppression activity of ClpP by β-lactones (Bottcher and Sieber 2008). Therefore, our objective was to identify small-molecule compounds with ClpP inhibitory activity to further enhance our arsenal for combating bacterial infections.

Coniferaldehyde (CA; 4-hydroxy-3-methoxycinnamaldehyde) is a phenolic compound present in the bark, leaves, and twigs of various edible plants, including cinnamomum cassia, salvia plebeia, and phyllanthus emblica (Kim et al. 2010; Yi et al. 2011; Zhang et al. 2016). This compound has been shown to possess anti-inflammatory, antioxidative, antiplatelet, and cytoprotective properties (Akram et al. 2016; Karamac et al. 2017). In this study, we present for the first time that CA is a promising candidate for further exploration and potential pharmaceutical development due to its ability to bind to intracellular ClpP and reduce MRSA virulence both in vitro and in vivo.

## Materials and methods

### Bacterial strains and culture conditions

Luria broth (LB) and LB agar plates were used for growth of *Escherichia coli* (*E. coli*), and Trypticase soy broth (TSB) and TSB agar plates were used for *S. aureus*. Kanamycin was used at 50 µg/mL for plasmid selection. Unless otherwise stated, all of the culture were grown aerobically at 37℃ with shaking, and growth was monitored at 600 nm with a spectrophotometer.

### Cloning, protein expression, and purification

The *clpP* gene fragment of *S. aureus* NCTC 8325-4 was amplified by PCR and inserted into prokaryotic expressing vector pET-28a (+). The recombinant plasmid was then transformed into *E. coli* BL21 cells, which were cultured under 37℃ until the OD_600 nm_ reached approximately 0.6. At this point, 0.1 mM IPTG was added to induce the expression of ClpP protein. After 24 h of induction at 16℃, the cells were harvested by centrifugation. The fusion protein was solubilized in the lysate buffer (PBS) using sonication, and the resulting supernatant was collected. The supernatant was then applied to a nickel affinity column packed with Ni Sepharose to purify the ClpP protein. After washed with PBS containing 10 mM imidazole or 20 mM imidazole, the ClpP was eluted with 250 mM imidazole for dialysis in dialysis buffer (150 mM NaCl and 20 mM Tris) overnight. Finally, the concentrations of ClpP proteins was determined by Nano drop.

### ClpP peptidase inhibition assay

The peptidase activity derived by ClpP for the different compounds were measured by the use of the fluorogenic substrate N-succinyl-Leu-Tyr-7-amido-4-methylcoumarin (Suc-Leu-Tyr-AMC) with the standard activity buffer (100 mM Hepes, pH 7.0, 100 mM NaCl) (Maurizi et al. 1994). 1 µM ClpP were incubated in ClpP activity buffer with varying concentrations of compounds or DMSO for control experiments for 10 min. Then, 200 µM Suc-Leu-Tyr-AMC were added up to 50 µL. Fluorescence was measured by a microplate reader (excitation 340 nm/emission 450 nm).

### CESTA assay

*S. aureus* NCTC 8325-4 was cultured at 37 °C in TSB and treated with DMSO or CA for 2 h. After resuspended in PBS buffer, and then exposed to heat treatment for 10 min at temperatures ranged from 25 °C to 62.8℃. After incubation, the cells were rapidly cooled, and the effects of heat exposure on protein stability were analyzed by western blot analysis. Briefly, the protein bands were incubated by mouse polyclonal anti-ClpP antibody overnight at 4℃ and then washed with TBST for three times. Following, the HRP-conjugated goat anti-mouse IgG(H + L) antibody (proteintech, China) was diluted at ration of 1:2000 in this assay. Finally, the relative intensities of the targeted proteins were visualized and quantified by Image J software.

### Creatine kinase (CK) activity assay

A mixture with 52 µg/mL creatine phosphate kinase (CPK), 20 µM ADP, 20 µM creatine phosphatase (CP) were added to ClpP activity treated with different concentrations of CA or DMSO and incubated at 37℃ for 10 min. Following this, 50 µL Kinase-Glo reagent (Promega) was added and incubated at 37℃ for another 10 min. Luminescence changes were measured using a SpaK 10 m (Tecan) plate reader after transferring the samples to a 96-well clear bottom plate.

### ClpP protease activity assay

Each ClpXP reaction contained 3 µM ClpP, 5 µM ClpX, and an ATP-regenerating system (1 mM ATP, 40 mM creatine kinase and 40 mM creatine phosphate). The GFP-tag SsrA was then added to each well and incubated at 37℃. The proteins were then analyzed by SDS-PAGE methods.

### Quantitative polymerase chain reaction (qPCR)

The total RNA was extracted from *S. aureus* NCTC 8325-4 using the TRIzol reagent kit (Mei5 Biotechnology Co.Ltd) and the total RNA was assessed with spectrophotometer. cDNA was synthesized from the total RNA using the StarScript Pro All-in-one RT Mix with gDNA Remover kit (Genstar). Real-time PCR was performed on the resulting cDNA using the Thermo Fisher Real-Time PCR System. The selected genes were analyzed using the primers were referred to previous research (Gao et al. 2018). The relative quantification of genes was determined using the 16SrRNA as the endogenous control.

### Hemolysis and proteolysis assay

*S. aureus* strains (NCTC 8325-4 (WT), ∆*clpP*, and ∆*clpP*/pYJ335::*clpP* (C-*clpP*) were cultured overnight in TSB medium. Hemolysis was tested on 5% sheep blood agar plates and proteolysis was assessed on LB agar plates containing 1% skimmed milk with different concentrations of CA. Stationary phase cultures of *S. aureus* (2.5 µL) were inoculated on the plates, and after overnight incubation at 37 °C for 12 h, the diameters of the zones around the bacterial colonies were measured.

For detecting neutralizing activity of CA against Hla, cultures of *S. aureus* NCTC 8325-4 or USA300 were grown for 8 h. The supernatant was harvested and mixed with 5% sheep red blood cells at a 1:1 ratio and different concentrations of CA. After incubation, the released hemoglobin in the supernatant was measured at 570 nm.

### Cytotoxicity study

The LDH assay was performed to evaluated the cytotoxicity of CA against Raw 264.7 or A549 cells. Briefly, the cells were seed into 96-well plates for 12 h and then treated with different concentrations of CA ranged from 4 µg/mL to 128 µg/mL at 37 °C for 6 h. After that, collected the culture supernatant at 1000 g, 10 min for LDH release detection with the optical densities at 492 nm and calculated cell viability based on its specification. The cells treated with DMSO or Triton X 100 were visualized with negative control or positive control respectively.

### Invasion assay

Overnight cultures of *S. aureus* strains (NCTC 8325-4, Δ*clpP*, and Δ*clpP*/pYJ335::*clpP*) with or without CA treatment were diluted and added to A549 cells at a multiplicity of infection (MOI) of 50. After 2 h of incubation at 37℃, the cells were washed to remove non-adherent bacteria, followed by the addition of fresh medium containing 50 µg/mL gentamicin to kill any remaining extracellular bacteria. The cells were then lysed in 1% saponin. and samples plated on TSB agar plates to determine the recovered colony-forming units (CFU).

### Cellular infections

To investigate the protective effects of CA against *S. aureus* infection on A549 cells, both LDH release assays and live/dead staining assays were conducted. A549 cells are infected with an overnight culture of *S. aureus* NCTC 8325-4, Δ*clpP* or USA300 at a multiplicity of infection of 100, with or without PCA treatment and centrifuged at 1000 g for 10 min. After 6 h of infection, the supernatants were mixed with LDH assay reagents to quantify the LDH release.

Additionally, the infected cells were subjected to live/dead staining using the Live/Dead Viability kits (Beyotime). Calcein-AM stains live cells green, while propidium iodide (PI) penetrates only dead or damaged cells, staining them red. After the staining procedure, the cells are examined under a fluorescence microscope.

### Stress assays

*S. aureus* strains (NCTC 8325-4, USA 300 and ∆*clpP*) was cultured at 37℃ and incubated with DMSO or CA for 2 h, and then the bacterial cultures were subjected to different environmental stress conditions.

For the oxidative stress assay, bacteria were treated with 0.5% NaClO or 1% H₂O₂ for 30 min after which the surviving cells were plated, and the survival rate was calculated relative to the DMSO control. To assess sensitivity to heat shock, cultures were incubated at 50℃ for 30 min, and then allowed to recover for 12 h before calculating the survival rate. In the acidic stress assay, bacteria were grown in medium containing 1 g/L tryptone, 5 g/L NaCl, and pH adjusted to 5.5 or 3.5. Following incubation, serial dilutions were plated onto TSB medium, and the bacterial concentration was determined to calculate the survival rate. Lastly, to evaluate the effect of the ClpP inhibitor under hypertonic conditions, cultures were grown in TSB medium with or without 10% NaCl for 6 h, with the survival rate calculated accordingly.

### Time killing assay

*S. aureus* (NCTC 8325-4 and Δ*clpP*) strains were diluted in TSB and cultured to OD_600nm_ of 0.6 at 37℃. The cultures were then diluted to 5 × 10^5^ CFU/mL in TSB and treated with tigecycline (0.2 µg/mL), CA (64 µg/mL) or their combination. Following this, all groups are incubated at 37℃, with samples taken at predetermined time intervals (0, 2, 4, 6, 8, and 12 h). At each time point, aliquots of the culture are removed and diluted appropriately before plating onto agar plates to determine viable counts by calculating CFU.

### Combined disk test

The combined disk test assay was performed to visualize the synergistic effect between CA and tigecycline. The overnight cultures were diluted with TSB to the OD_600 nm_=0.1. And then, the strains were poured into the plate with 32 µg/mL or 64 µg/mL CA. The disks containing the 0.2 µg/mL tigecycline were placed the center of the plate. After incubation for 24 h at 37℃, the inhibition zone was recorded.

### Live/dead bacteria staining

*S. aureus* strains (NCTC 8325-4 and Δ*clpP*) were diluted in TSB medium and then incubated with tigecycline (0.2 µg/mL), CA (64 µg/mL) or their combination at 37℃ for 6 h. After centrifuge, the bacteria were resuspended to 0.5 at OD_600 nm_ with PBS. The bacterial suspension was mixed with the dyes using the LIVE/DEAD BacLight Bacterial Viability Kit (Invitrogen) according to the manufacturer’s instructions. After incubation, the samples are observed under the fluorescence microscope.

### Scanning electron microscopy (SEM)

Overnight cultures of *S. aureus* (NCTC 8325-4 and Δ*clpP*) strains were diluted in fresh TSB medium and incubated at 37℃ with tigecycline (0.2 µg/mL), CA (64 µg/mL) or a combination of both for 3 h. The bacterial cells were then collected, washed with PBS, and fixed in 2.5% glutaraldehyde overnight at 4℃. After fixation, the samples underwent dehydration using increasing concentrations of ethanol (30%, 50%, 70%, 90%, and absolute 100%), followed by critical-point drying to prevent osmotic damage during the imaging process. Dried samples were gold-coated and analyzed under the scanning electron microscope.

### *Galleria mellonella* infection model

The *Galleria mellonella* infection model was performed to explore the protective effect of CA against *S. aureus* USA300. The 200 mg-230 mg *Galleria mellonella* was infected by *S. aureus* USA300 at a dose of 1 × 10^7^ CFU per larva via the leg injection. After infected for 2 h, the *Galleria mellonella* were treated with 25 mg/kg or 50 mg/kg CA once. The survival rate of *G. mellonella* was monitored every 6 h for 30 h.

### Circular dichroism (CD) spectra analysis

The CD spectra of ClpP with or without CA were analyzed over a wavelength range of 190 nm to 250 nm using a CD spectropolarimeter, with the temperature controlled to maintain consistency across measurements. The secondary structure of the protein was analyzed using the BeStSel web server.

### Molecular dynamics (MD) simulations

The PDB code for the initial 3D structure of ClpP was 7wid, and the standard docking procedures of CA and ClpP were performed using AutoDock Vina, following docking, selected poses were subjected to molecular dynamics (MD) simulations using GROMACS software. The complex was solvated in a water box, and appropriate Na^+^ were added to neutralize the system. The MD simulations were performed at physiological temperature and pressure, while monitoring the root-mean-square deviation (RMSD), root-mean-square fluctuation (RMSF), and hydrogen bonds to analyze the stability and interactions within the complex. The binding free energy between the protein and the ligand was calculated using the molecular mechanics Poisson Boltzmann surface area (MM-PBSA) method.

### Mutagenesis

The primers were used to amplify the entire plasmid pET28a-ClpP for generation of site-specific mutations. The mutated products were extracted and transformed into DH5α competent bacterial cells. And the mutated sequences of the *clpP* gene were verified and confirmed by DNA sequencing. Variant ClpP proteins were expressed in *E. coli* BL21(DE3) and purification with Ni-NTA affinity chromatography methods, which was detailed describe in “2.2 Cloning, protein expression, and purification” section. Moreover, the mutant proteins were also purified for further enzymatic cleavage assays using Suc-LY-AMC as the substrate.

### Fluorescence quenching analysis

The binding constants (Ka) between CA and binding sites on ClpP, M31A, I30A, L25A, and G33A were determined using a fluorescence-quenching method at excitation wavelength of 290 nm and emission wavelength of 500 nm following previously described methods (Xu et al. 2022).

### Pneumonia model experiment

Female C57BL/6J mice (6–8 weeks old) were obtained from Liaoning Changsheng Biotechnology Co., Ltd. Mouse experiments were approved by the Animal Care and Use Committee of Jilin University (SY202412062). *S. aureus* strain NCTC 8325-4 and ∆*clpP* were cultured to the mid-log growth phase in TSB, washed with PBS, resuspended in sterile saline at 1 × 10^7^ CFU/100 µL for the sublethal pneumonia model.

The mice were infected with *S. aureus* by nasal drip method. (50 µL/mouse) and randomized into three groups (solvent control, 15 mg/kg CA, and 30 mg/kg CA). Another group received the same volume of *S. aureus* ∆*clpP* and this served as a control. One hour after infection, mice were treated with subcutaneous injection of the designated concentrations of compound CA (15 mg/kg or 30 mg/kg) or control solvent every 12 h. For the sublethal pneumonia model, mice from each group were euthanized at 48 h post infection, and the lungs were fixed with 4% formaldehyde for the histological assessment. Then, the bacterial counts were determined from bronchoalveolar lavage fluid (BALF) and lung homogenates on TSB agar plate medium. In addition, cytokines (TNF-α, IL-6 and IL-1β) were assessed using ELISA kits (BioLegend).

### Statistical analyses

All experiments in this study were performed in triplicates unless specified otherwise. Students t test in GraphPad Prism Software 8.0 was used for all statistical analyses. And all data are showed as mean ± SD. Asterisks indicate statistically significant differences by t-test (*, *p* < 0.05, **, *p* < 0.01, ns, not significant).

## Results

### Identification of CA as an ClpP inhibitor

**Fig. 1:**
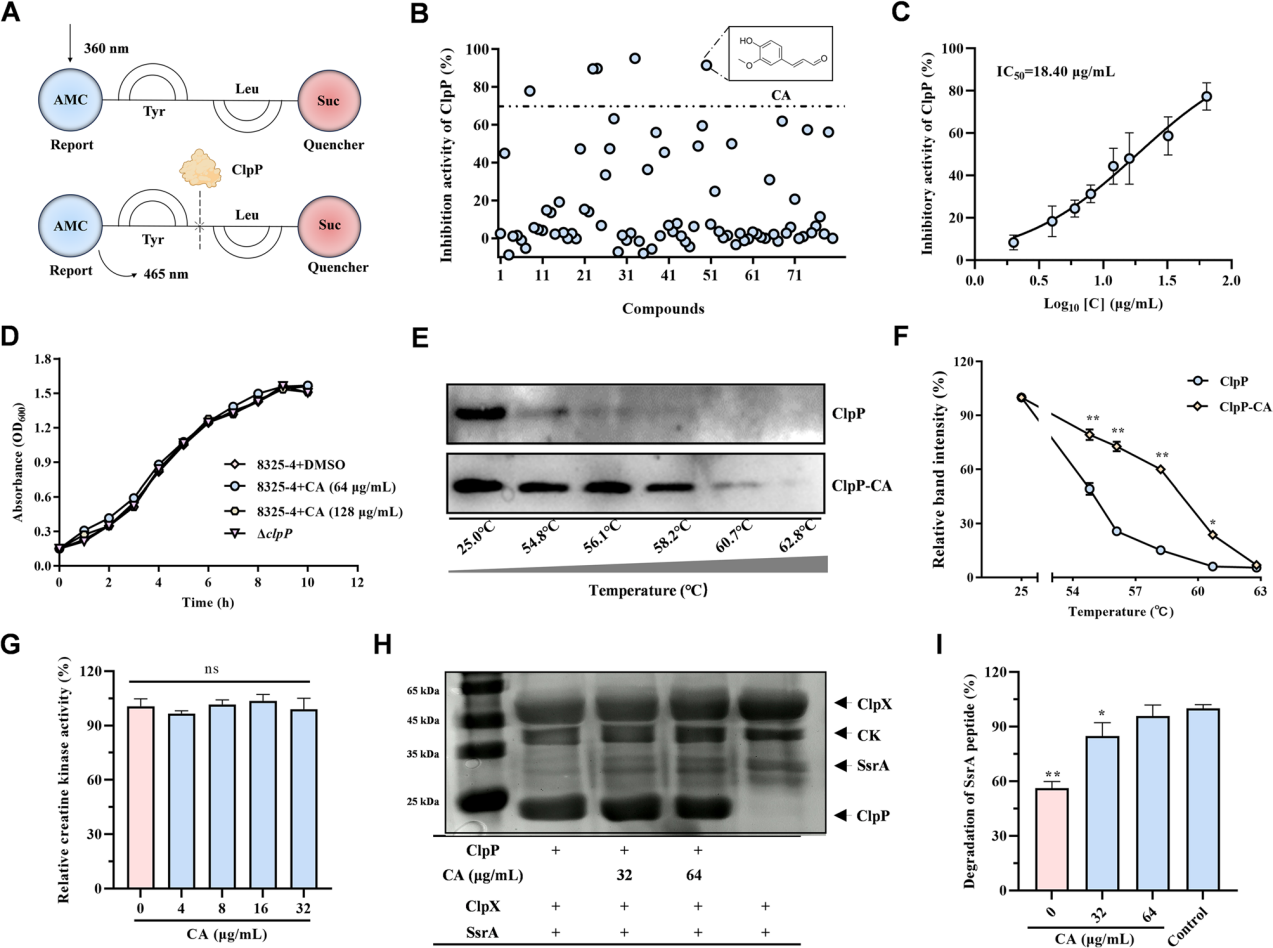
CA was discovered as an ClpP inhibitor in *S. aureus.***A** A schematic diagram of natural compound inhibitor screening process, showing the ability of the ClpP enzyme to cleave the Suc-LY-AMC substrate. **B** Effectiveness of different compounds in inhibiting ClpP. Compounds showing more than 70% inhibition were identified as potential inhibitors. **C** CA inhibited the ClpP hydrolytic ability against SUC-LY-AMC oligopeptide. **D** The growth curves of *S. aureus* treated with or without CA. **E** and **F** Western blot lines (**E**) and optical density analysis (**F**) of CETSA assay showing the heat stability of ClpP with or without CA. **G** - **I** CA did not affect the creatine kinase activity in the SsrA dagration reaction (**G**), but inhibited the ClpP’s ability to hydrolyze SsrA in the presence of ClpX at the concentrations of 32 µg/mL or 64 µg/mL (**H**). And the relative degradation of SsrA peptide was also analyzed (**I**)

To identify the novel inhibitors of ClpP, we evaluated the ClpP-catalyzed hydrolysis of the SLY-AMC peptide (schematic diagram as shown in Fig. 1A) using an in-house library of natural origin compounds (Fig. 1B). Notably, the compound CA exhibited the most significant inhibition, with an IC_50_ of 18.40 µg/mL (Fig. 1C) and had no effect on the bacteria growth (Fig. 1D). To explore the direct interaction between CA and ClpP, we performed thermal stability analysis using CESTA. The results indicated that ClpP treated with increasing concentrations of CA remained more stable at 58.2 °C, in contrast to the control, suggesting a strong affinity between CA and ClpP **(**Fig. 1E and F). ClpP can degrade SsrA in the help of ClpX in an ATP-dependent process derived by creatine kinase (CK) (Gottesman et al. 1998; Kim et al. 2000). Furthermore, we assessed the impact of CA on the degradation of SsrA peptide sequence by ATPases activate ClpP. As shown in Fig. 1G and H, CA did not interfere the ATP regeneration system derived by creatine kinase but inhibited the degradation activity of ClpP against SsrA in Fig. 1H. And the relative degradation of SsrA peptide was shown in the Fig. 1I. These findings demonstrated that CA effectively inhibits the proteolytic activity of the ClpP without hindering ATP production, which indicated that CA is an effective ClpP inhibitor.

### Attenuation of *S. aureus* virulence by CA in vitro

**Fig. 2:**
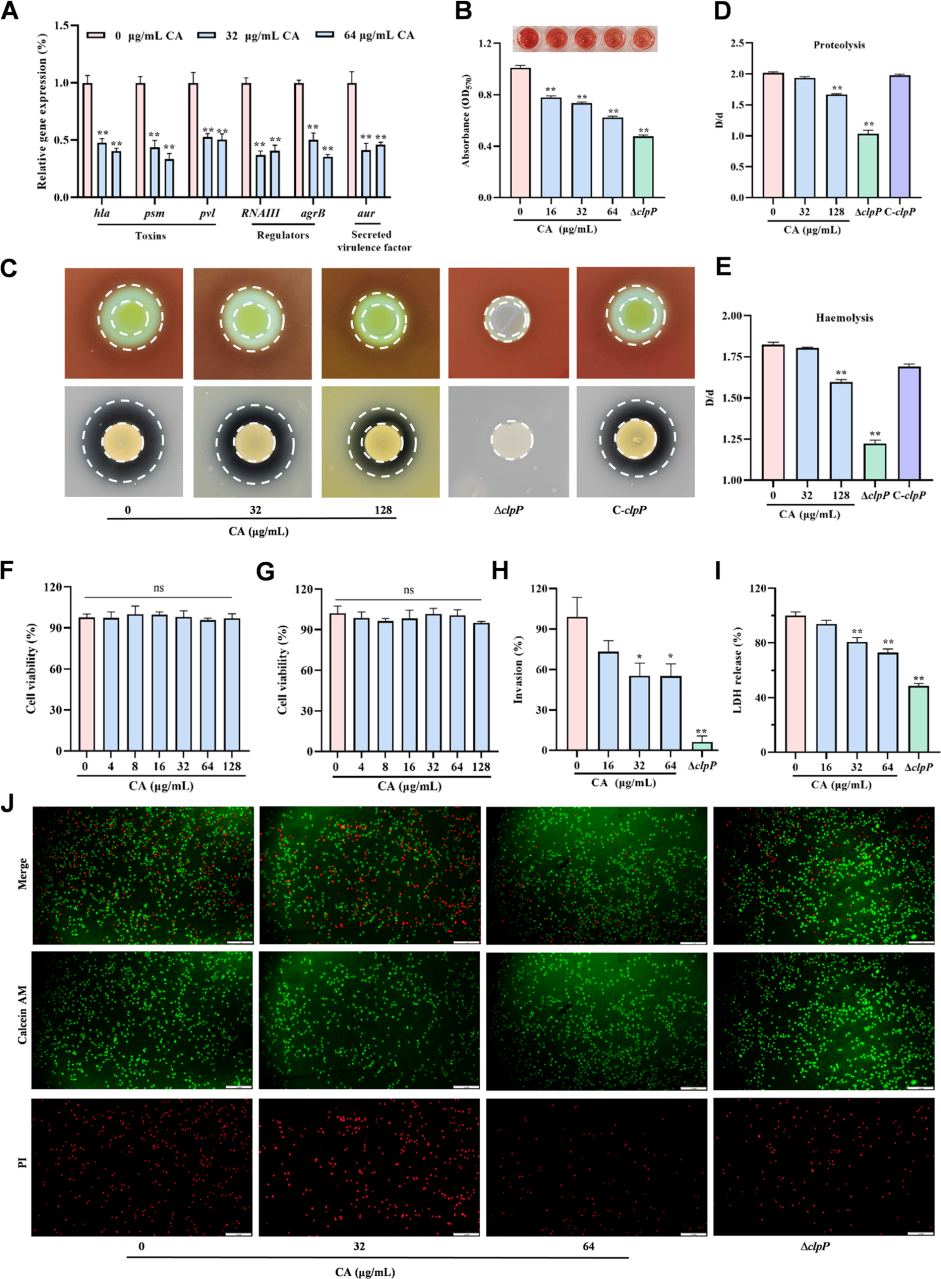
CA reduced the virulence of *S. aureus in vitro*. **A** The virulence related gene expression level in *S. aureus* was determined by real time PCR with the treatment of CA (32 µg/mL or 64 µg/mL). **B**-**E** CA inhibited the hemolytic activity of culture supernatant and proteolytic ability of *S. aureus* in 1% skim milk agar plates (**B** and **C**) Moreover, the ratio of halo diameter to colony diameter (D/d) reflected the hemolysis and extracellular proteolysis ability of strains was shown in **D** and **E**. **F** and **G** CA showing no cytotoxicity in Raw264.7 and A549 cells at less than 128 µg/ml. **H** and **I** Reduced the bacterial intracellular colonization number (**H**) and protect the cells from toxicity induced by *S. aureus* infection (**I**). **J**: Visualizing the protective of CA at 32 µg/mL or 64 µg/mL against *S. aureus* infection using the Live/dead cell staining kit

The successful inhibition of ClpP by CA raised the question whether the compound could also impair its natural function, leading to a reduced production of virulence factors in ∆*clpP* mutant *S. aureus* strain. As shown in Fig. 2A, the major virulence factor genes of *hla*, *pvl*, *psm* and *agrA* were significantly downregulated by CA in *S. aureus.*

The pathogenicity of *S. aureus* requires a large number of cell surface-associated and secreted proteins. The deficiency of *clpP* leads to dysregulation of the expression of toxicity-related proteins, highlighting the role of *clpP* in the pathogenic mechanism of *S. aureus* (Frees et al. 2005). Among the secreted proteins include extracellular proteases and α-hemolysin (Frees et al. 2012). The collected *S. aureus* NCTC8325-4 culture supernatants were cultured with erythrocytes to assess the level of secreted Hla. A dose-dependent inhibition effect of CA on hemolysis against erythrocytes was observed (Fig. 2B). Agar plate-based assays with 5% sheep blood further confirmed inhibition of hemolysis in *S. aureus* for CA (Fig. 2C). In addition, corresponding assays on 1% skim milk agar plates revealed that CA inhibited extracellular proteolysis (Figs. 2C). The ratio of halo diameter (D) to colony diameter (d) (D/d) reflected the hemolysis and extracellular proteolysis ability of strains. Our D/d value indicated that CA possessed the significantly inhibition effect of hemolysis and proteolysis at above the 128 µg/mL (Figs. 2D and E).

The surface-associated proteins possessed by *S. aureus* to bind to host fibrinogen, fibronectin, collagen, and von willebrand factor, thus enabling the bacteria to colonize and establish a focus of infection. Thus, the effect of CA on invasion of *S. aureus* to epithelial cells was further evaluated. The results indicated that CA exhibited no significant cytotoxicity to Raw 264.7 and A549 cells within the effective concentration range (Fig. 2F and G), but the invasion rate of *S. aureus* is significantly reduced following treatment with CA (Fig. 2H**)**, and further damage to the cell membrane was also inhibited by CA according to the live/dead assay (green: live, red: dead) and the lactate dehydrogenase (LDH) assay **(**Fig. 2I and J). Overall, CA can affect virulence of *S. aureus* by affecting the specific virulence factors transcription.

### Reduction of environmental tolerance by CA of *S. aureus*

**Fig. 3:**
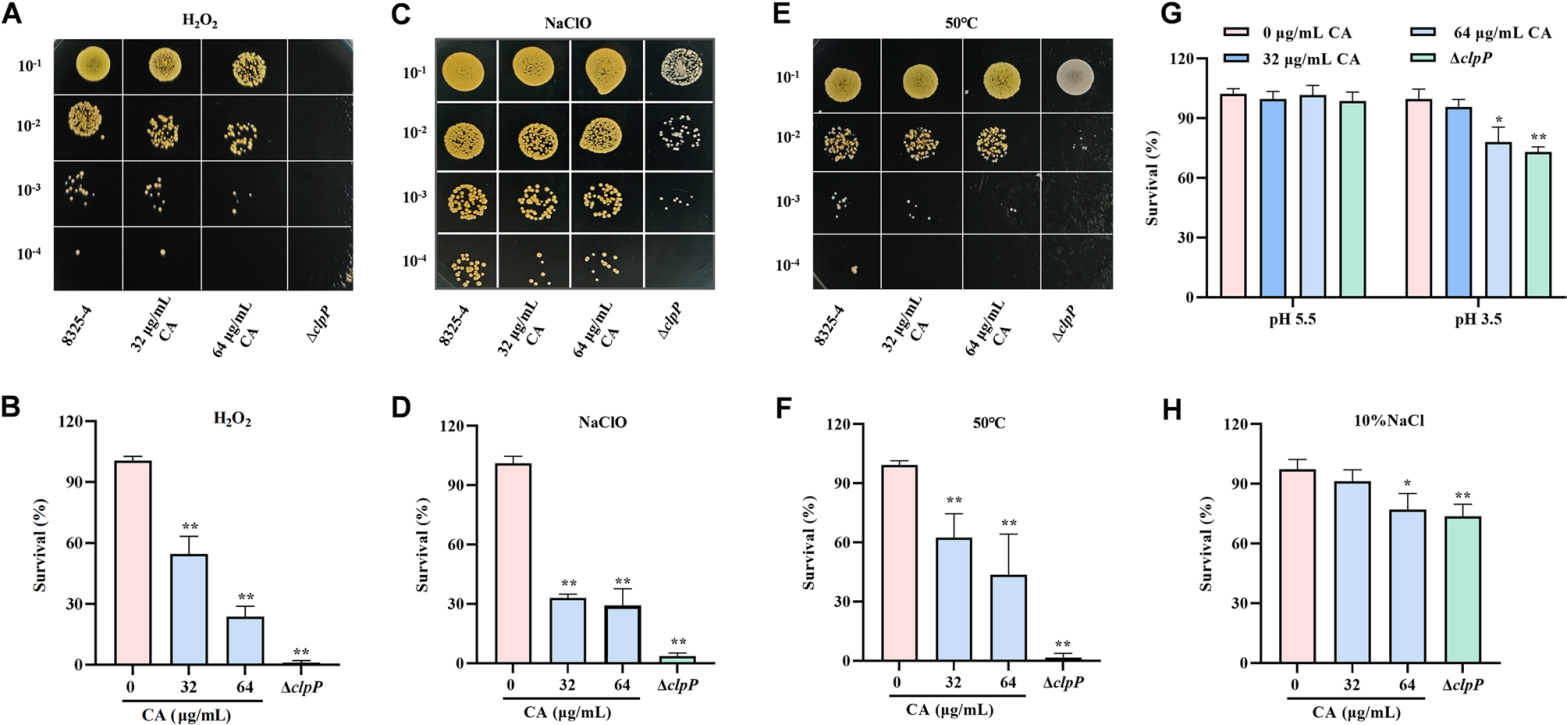
CA weaken the environmental tolerance of *S. aureus in vitro*. **A**-**F** The survival of indicated strains treated with CA above 64 µg/mL was spotted on the LB agar plate in 10-fold serial dilution after H_2_O_2_ (**A**), NaClO (**C**) or heat (**E**) stress. Moreover, the corresponding colony count results was showing the **B**, **D** and **F**. **G** and **H** The survival rate of strains at different culture condition (pH 3.5, pH 5.5 and 10% NaCl) was determined by plate count

Based on previous research, the ClpP is involved in not only the regulation of protein expression and secretion but also the degradation of misfolded proteins under stress conditions, including temperature shifts, pH changes, and exposure to reactive oxygen species, generated by host phagocytes (Gottesman 2003; Michel et al. 2006). Therefore, we further investigated whether the suppressed ClpP activity by CA is correlation to the reduced stress tolerance of *S. aureus.* The results indicated that the inhibition of ClpP by CA rendered the cells sensitive to hydrogen peroxide and NaClO (Fig. 3A-D**)**, and the growth of *S. aureus* treated with CA was impaired in high temperatures, high salt, and low pH conditions experiments (Fig. 3E-H**)**. Thus, we demonstrated that the role of CA in reducing the tolerance of *S. aureus* towards stress conditions.

### Enhancement of sensitivity to Tigecycline by CA of *S. aureus*

**Fig. 4:**
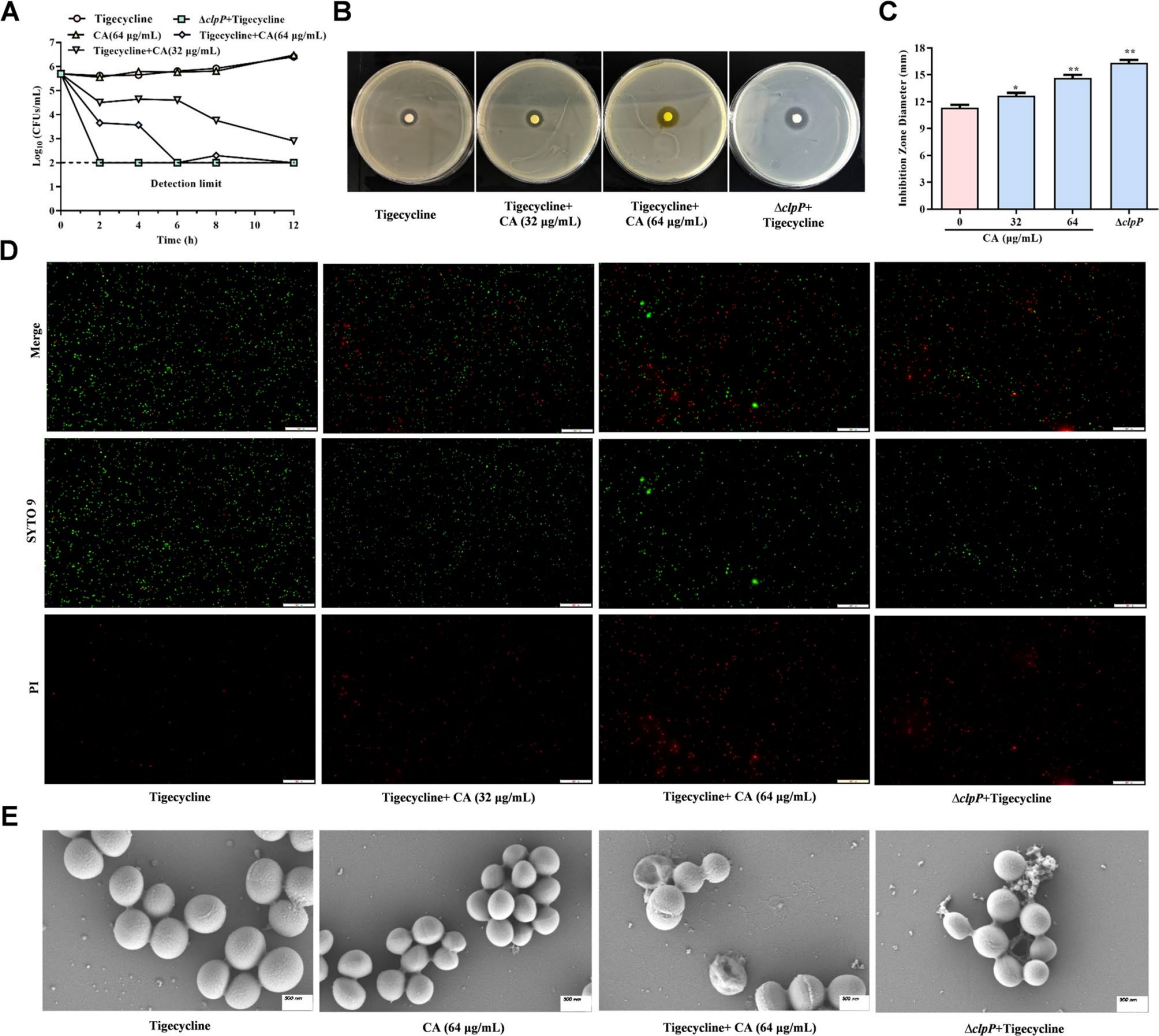
CA enhanced the bactericidal efficiency of tigecycline against *S. aureus in vitro*. **A** Time-killing curves evaluation of CA (64 µg/mL), tigecycline (0.2 µg/mL) or combinations on *S. aureus* strains every 2 h for 12 h. **B** and **C** Eye view of combining drug susceptibility test between CA and tigecycline (**B**). Corresponding inhibition zone diameter was also recorded (**C**). **D** and **E** The cell permeability and death treated with CA (64 µg/mL), tigecycline (0.2 µg/mL) or combinations were analyzed by Live/Dead bacteria staining and Scanning electron microscopy

It has been reported that truncation mutation of *clpP* confers susceptibility to protein synthesis inhibitor antibiotics (Shoji et al. 2011). Thus, we further determined whether the combination of CA and tigecycline has an effective bactericidal effect in vitro. The combination of CA with tetracycline can reduce the number of tested bacteria to the limit of detection within 12 h compared with monotherapies (Fig. 4A). Similarly, the combination of CA and tigecycline increased the diameter of the inhibition zones compared to the non-combination group (Fig. 4B, C). Consistent with these results, in the samples treated with monotherapy or without treatment, most bacteria survived with a normal cellular morphology, however, combination therapy resulted in increased cell permeability and death, which was evidenced by significantly more red-stained bacteria observed with the Live/Dead BacLight bacterial viability kit and significantly damaged bacteria examined under SEM (Fig. 4D, E). Taken together our findings indicate that CA can effectively restore the bactericidal effect of tigecycline by inhibiting ClpP.

### CA exhibited an anti-infection ability against *S. aureus* USA300

**Fig. 5:**
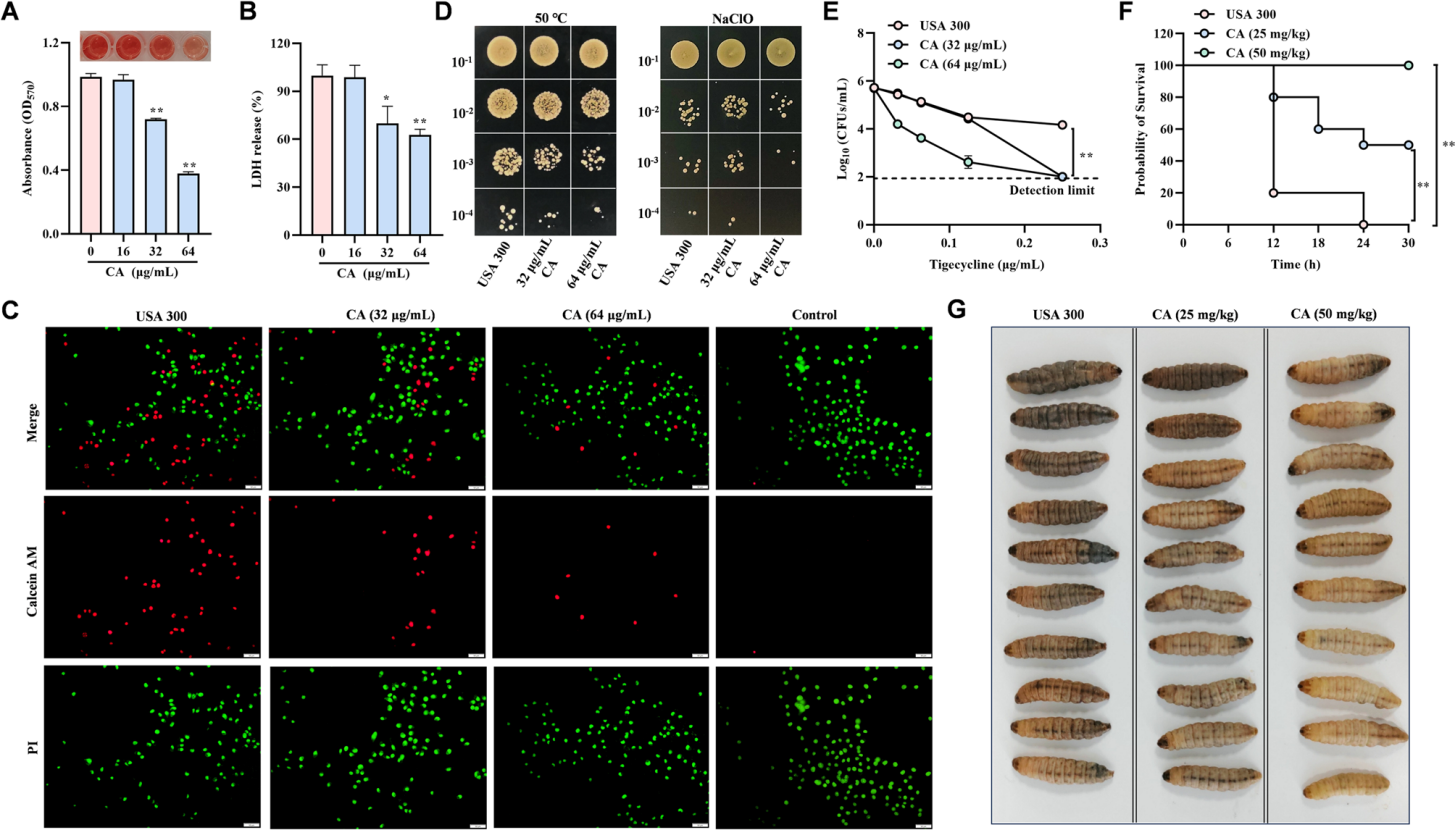
CA exhibited an anti-infection ability against *S. aureus* USA300 (**A**) CA inhibited the culture supernatant hemolytic activity of *S. aureus* USA300 at above 32 µg/mL. **B** The LDH release determination in cellular infections model induced by *S. aureus* USA300. **C** Live/dead cells staining assay for visualizing the cells injury in cellular infections model. The green or red marked cells were shown live or dead cells respectively. **D** and **E** CA weaken the environmental tolerance of *S. aureus* USA300 against heat, NaClO (**D**) and tigecycline stress (**E**). **F** and **G** *Galleria mellonella* infection model was performed to evaluated the protective effect of CA against *S. aureus* USA300. The survival rate and state were shown in **F** and **G** respectively

In order to explore the anti-infection ability of CA against clinically isolated *S.aureus* USA300, we conducted several important phenotype experiments including hemolysis test, cellular infections, stress assay, time killing assay in vitro and *Galler mellonella* infection model in vivo. As shown in Fig. 5A, CA could inhibit the hemolytic activity of *S. aureus* USA300 culture supernatant at above 32 µg/mL. 32 µg/mL or 64 µg/mL CA also exhibited a protective effect in cellular infections, showing the decreased LDH release in Fig. 5B and injured A549 cells (red fluorescence marked cells) in Fig. 5C. Moreover, CA enhanced the susceptibility of *S. aureus* USA300 against heat, NaClO and tigecycline stress in Fig. 5D and E. Importantly, 25 mg/kg or 50 mg/kg CA improved the *Galler mellonella* survival rate in Fig. 5F, compared with *S. aureus* USA300 infected group. And the survival status was shown in Fig. 5G at 30 h. Above all, these results indicated that CA exhibited an anti-infection ability against *S. aureus* USA300.

### Identification of the mechanism of the interaction between CA and ClpP

**Fig. 6:**
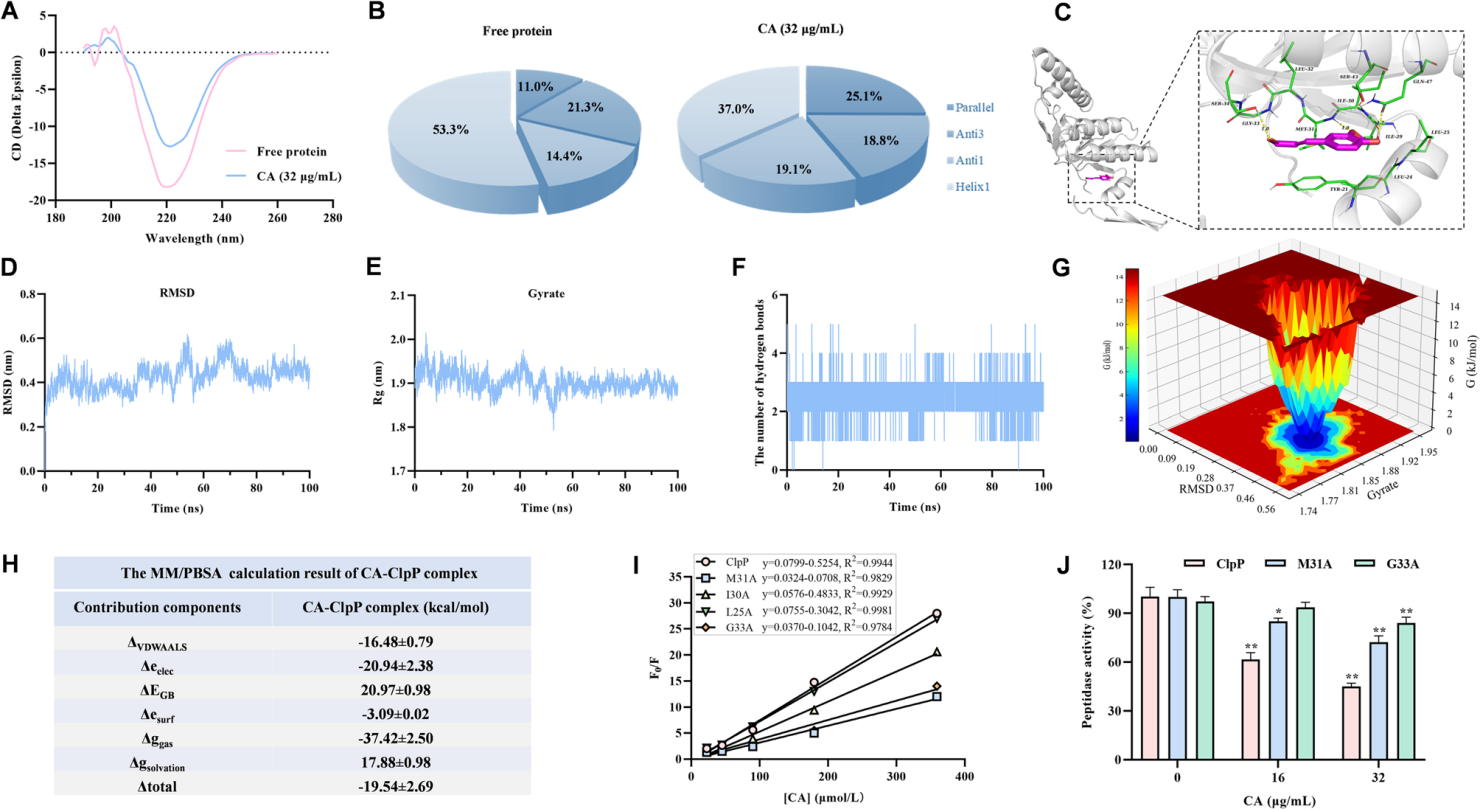
Identification of the mechanism of the interaction between CA and ClpP. **A** and **B** Secondary structure changes of ClpP treated with 32 µg/mL CA. **C**-**H** CA and ClpP docking complex was created (**C**), and RMSD (Root Mean Square Deviation) (**D**), Rg (Radius of Gyration) (**E**), Hydrogen bond (**F**), FES (Free energy surface) (**G**) and RBFE (Relative binding free energy) (**H**) date were recorded. **I** and **J** The binding constants (Ka) of the pivotal mutants between CA was calculated by fluorescence quenching assay. Moreover, ClpP and mutant peptidase activity test were also test with 16–32 µg/mL CA

The stability of ClpP treated with CA was investigated by circular dichroism spectra. The results showed that changes in secondary structures occurred when CA were added to ClpP, the α-helix content decreased and β-sheet content increased (Fig. 6A, B).

Molecular docking was employed to further explore the binding interaction between CA and ClpP. A visual analysis of the ClpP-CA docking complex was conducted using PyMol2 and Discovery Studio Visualizer software to generate a 3D interaction map (Fig. 6C). To validate the docking results, molecular dynamics simulations were performed. Analysis of RMSD, Rg, hydrogen bonds, free energy surfaces (FES), and relative binding free energy (RBFE) indicated a compact protein structure and reduced solvent-accessible surface area after 70 ns (Fig. 6D-H). We concluded that, the protein tightly enveloped the drug small molecule in the presence of a certain number of hydrogen bonding interaction forces at steady state.

Mutation experiments and fluorescence quenching analyses were performed to illustrate CA’s detailed action on ClpP. The binding constants (Ka) of the mutants were significantly lower than that of ClpP (Fig. 6I). Additionally, CA’s inhibitory effect on ClpP’s peptidase activity was diminished in the M31A and G33A mutants (Fig. 6J). These observations indicated that the residues M31 and G33 are required for the engagement of CA with ClpP.

### Anti-infective effects of CA against *S. aureus in vivo*

**Fig. 7:**
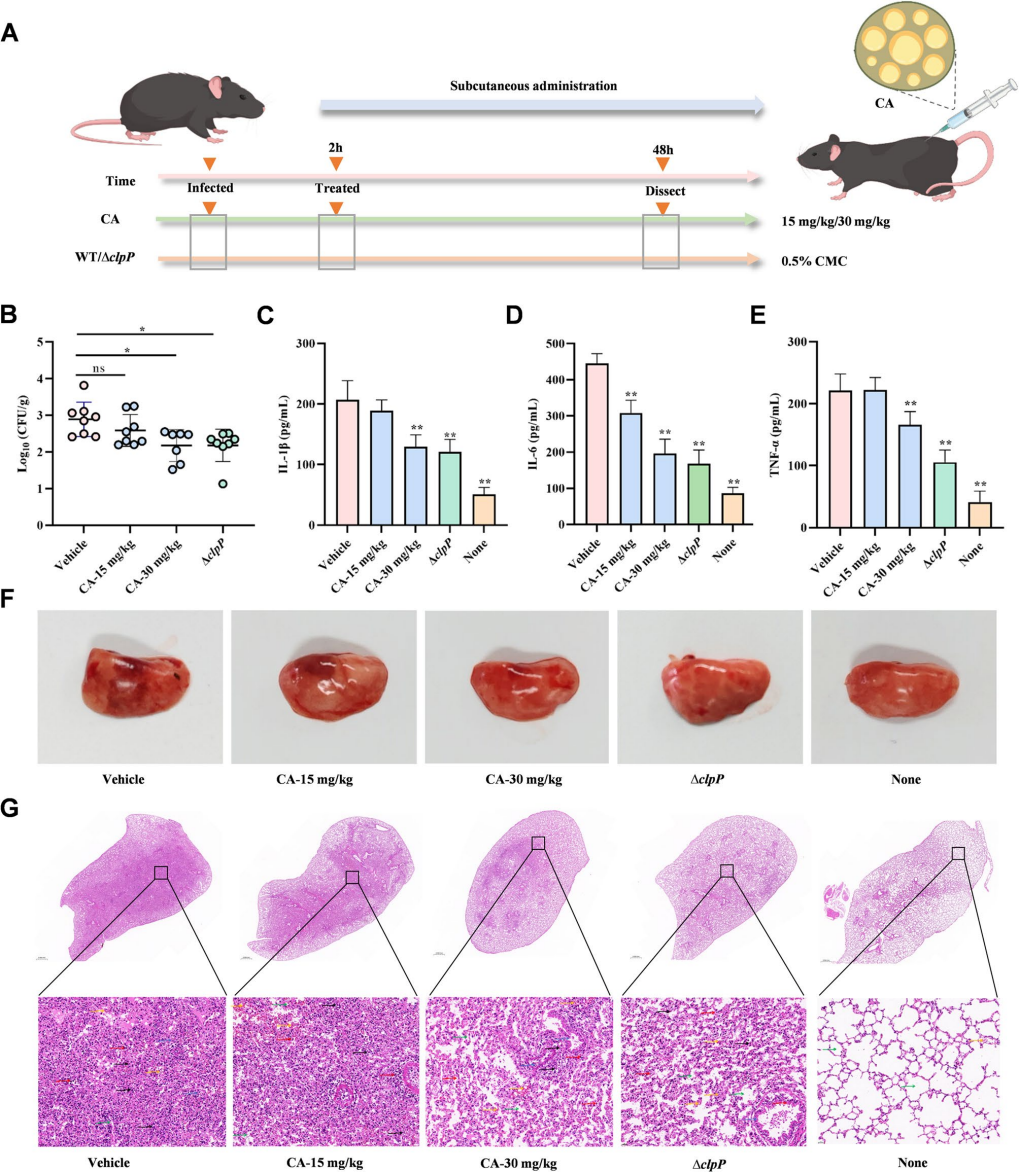
CA relieved mouse pneumonia injury induced by *S. aureus in vivo*. **A** Schematic diagram of the mouse pneumonia model infected with *S. aureus*. Mice were infected with *S. aureus* 1 × 10^7^ CFU/mouse and treated with CA (15 mg/kg or 30 mg/kg). Lung tissue was collected for bacterial load (**B**), cytokines expression level evaluation (**C**-**E**) and histopathological examination (**F** and **G**). Green arrow: healthy alveolar space; yellow arrow: pulmonary hemorrhage; blue arrow: bronchiole; red arrow: inflammatory cells; black arrow: neutrophils

To investigate the protective efficacy of CA against *S. aureus*, murine models of MRSA pneumonia infection were established. Mice were intratracheally instilled with live *S. aureus*, and lung tissues were collected after 2 days to enumerate the bacterial loads (schematic diagram as shown in Fig. 7A). As shown in the Fig. 7B, both the CA-treated group (30 mg/kg) and the ∆*clpP* group exhibited a significant reduction in the colony-forming units of *S. aureus* in the lungs compared to the vehicle group, suggesting that CA effectively reduced bacterial colonization.

To further explore the therapeutic benefits of CA in treating acute *S. aureus* pneumonia, we investigated lung inflammation and cytokine production in mice. The levels of inflammatory cytokines, including TNF-α, IL-6, and IL-1β, were markedly lower in the bronchoalveolar lavage fluid (BALF) of both CA-treated (30 mg/kg) and ∆*clpP* animals **(**Fig. 7C-E). Meanwhile, mice treated with solvent developed more obvious pneumorrhagia than mice treated with 30 mg/kg CA at 48 h postinfection (Fig. 7F**)**. Histopathological examination through hematoxylin and eosin (HE) staining revealed less inflammatory cell infiltration and preserved lung architecture in both the CA-treated and ∆*clpP* groups, as opposed to the untreated group, which showed extensive infiltration, edema, and alveolar damage (Fig. 7G**)**. Taken together, these results indicated that CA treatment relieved inflammation and cytokine levels, which show an excellent protective effect.

## Discussion

*S. aureus* obtained nearly all available antibiotics, highlighting the need for new therapeutic targets and innovative strategies to address *S. aureus*-related infections. ClpP and ClpX are essential for regulating the expression of important virulence factors and protein transcription in this bacterium (Mei et al. 1997). To coordinate the release of these viluence, *S. aureus* strains employ a complex global regulatory network (Gimza et al. 2019). The ClpXP protease system promotes the transcription of *mgrA* and *agr*, which increases the levels of RNAIII. This, in turn, suppresses the expression of the repressor of toxins (Rot) and promotes the production of multiple toxins, including the pore-forming hemolysin alpha-toxin (Jelsbak et al. 2010; Aljghami et al. 2022). Through this intricate regulatory mechanism, ClpP and ClpX are integral to the pathogenicity of *S. aureus*, ensuring that virulence factors are expressed in a coordinated manner to optimize infection. Furthermore, the loss of clpP results in a complete transcriptional depression of genes regulated by the *ctsR* and *hrcA* heat shock regulon, as well as a partial depression of genes associated with the oxidative stress response, metal homeostasis, and SOS DNA repair, which are controlled by regulatory proteins such as PerR, Fur, MntR, and LexA (Michel et al. 2006). This alteration in gene expression underscores the significant role of ClpP in maintaining the balance of stress responses and homeostasis in *S. aureus*.

The exploration of inhibitors targeting ClpP has garnered significant attention due to their critical role in bacterial virulence and pathogenesis (Gaillot et al. 2000; Michalik et al. 2012; Zhao et al. 2016). However, the quest for effective caseinolytic protease inhibitors is fraught with challenges. β-Lactones has been displayed ClpP inhibition in a variety of pathogens such as *P. alciparum* or *M. tuberculosis* (Rathore et al. 2010; Compton et al. 2013). Despite promising results in vitro, the clinical development of cyclic esters has been hampered by reduced plasma stability due to hydrolysis and their generally low selectivity (Hackl et al. 2015). Bortezomib, a known inhibitor of the 26 S proteasome, has also been shown to inhibit ClpP1P2 (Moreira et al. 2015). However, its high cost, short half-life, and poor pharmacokinetics limited its effectiveness in treating M. tuberculosis. In this study, we demonstrate that the natural compound CA reduces the virulence of *S. aureus* by inhibiting ClpP. One significant advantage of CA is its status as a legally approved food flavoring (Panossian et al. 2001; Choi et al. 2017; Dong et al. 2020). Analysis of CA levels in mice following oral administration (0.2 mmol/kg) indicated rapid absorption, peaking at around 1 h. Importantly, CA has a favorable safety profile (Oral LD_50_: mouse, 300 mg/kg; rabbit, 3200 mg/kg; rat, 980 mg/kg), which is well within the effective range tested here (Mei et al. 1997). Thus, CA has the potential to be a promising candidate for further clinical evaluation as a therapeutic agent against *S. aureus* pneumonia.

## Conclusion

We found CA could bind endogenous ClpP in *S. aureus* cells and exhibited significant efficacy in attenuating *S. aureus* virulence. Moreover, the persistence of *S. aureus* treated with CA under conditions of tigecycline exposure, as well as heat, oxidative stress, and osmotic pressure were decreased. Importantly, CA attenuated mice pneumonic injury induced by *S. aureus in vivo*. This work holds great potential to inspire promising strategies for treating MRSA infections and contributes to the development of potent synthetic antibiotic scaffolds with minimal cytotoxicity to the host.

## Data Availability

No datasets were generated or analysed during the current study.

## References

[CR1] Akram M, Kim KA, Kim ES, Shin YJ, Noh D, Kim E, Kim JH, Majid A, Chang SY, Kim JK, Bae ON. Selective Inhibition of JAK2/STAT1 signaling and iNOS expression mediates the anti-inflammatory effects of coniferyl aldehyde. Chem Biol Interact. 2016;256:102–10. 27378624 10.1016/j.cbi.2016.06.029

[CR2] Aljghami ME, Barghash MM, Majaesic E, Bhandari V, Houry WA. Cellular functions of the ClpP protease impacting bacterial virulence. Front Mol Biosci. 2022;9:1054408. 36533084 10.3389/fmolb.2022.1054408 PMC9753991

[CR3] Bottcher TSieber SA. Beta-lactones as specific inhibitors of ClpP attenuate the production of extracellular virulence factors of Staphylococcus aureus. J Am Chem Soc. 2008;130(44):14400–1. 18847196 10.1021/ja8051365

[CR4] Brotz-Oesterhelt HVorbach A. Reprogramming of the caseinolytic protease by ADEP antibiotics: molecular mechanism, cellular consequences, therapeutic potential. Front Mol Biosci. 2021;8:690902. 34109219 10.3389/fmolb.2021.690902 PMC8182300

[CR5] Chambers HFDeleo FR. Waves of resistance: Staphylococcus aureus in the antibiotic era. Nat Rev Microbiol. 2009;7(9):629–41. 19680247 10.1038/nrmicro2200 PMC2871281

[CR6] Choi SK, Mun GI, Choi E, Kim SY, Kwon Y, Na Y, Lee YS. The conjugated double bond of coniferyl aldehyde is essential for heat shock factor 1 mediated cytotoprotection. J Nat Prod. 2017;80(8):2379–83. 28737916 10.1021/acs.jnatprod.7b00126

[CR7] Compton CL, Schmitz KR, Sauer RT, Sello JK. Antibacterial activity of and resistance to small molecule inhibitors of the ClpP peptidase. ACS Chem Biol. 2013;8(12):2669–77. 24047344 10.1021/cb400577b PMC4287380

[CR8] Dong Y, Stewart T, Bai L, Li X, Xu T, Iliff J, Shi M, Zheng D, Yuan L, Wei T, Yang X, Zhang J. Coniferaldehyde attenuates alzheimer’s pathology via activation of Nrf2 and its targets. Theranostics. 2020;10(1):179–200. 31903114 10.7150/thno.36722 PMC6929631

[CR9] Frees D, Qazi SN, Hill PJ, Ingmer H. Alternative roles of ClpX and ClpP in Staphylococcus aureus stress tolerance and virulence. Mol Microbiol. 2003;48(6):1565–78. 12791139 10.1046/j.1365-2958.2003.03524.x

[CR10] Frees D, Sorensen K, Ingmer H. Global virulence regulation in Staphylococcus aureus: Pinpointing the roles of ClpP and ClpX in the sar/agr regulatory network. Infect Immun. 2005;73(12):8100–8. 16299304 10.1128/IAI.73.12.8100-8108.2005 PMC1307069

[CR11] Frees D, Andersen JH, Hemmingsen L, Koskenniemi K, Baek KT, Muhammed MK, Gudeta DD, Nyman TA, Sukura A, Varmanen P, Savijoki K. New insights into Staphylococcus aureus stress tolerance and virulence regulation from an analysis of the role of the ClpP protease in the strains newman, COL, and SA564. J Proteome Res. 2012;11(1):95–108. 22112206 10.1021/pr200956s

[CR12] Frieri M, Kumar K, Boutin A. Antibiotic resistance. J Infect Public Health. 2017;10(4):369–78. 27616769 10.1016/j.jiph.2016.08.007

[CR13] Gaillot O, Pellegrini E, Bregenholt S, Nair S, Berche P. The ClpP Serine protease is essential for the intracellular parasitism and virulence of Listeria monocytogenes. Mol Microbiol. 2000;35(6):1286–94. 10760131 10.1046/j.1365-2958.2000.01773.x

[CR14] Gao P, Ho PL, Yan B, Sze KH, Davies J, Kao RYT. Suppression of Staphylococcus aureus virulence by a small-molecule compound. Proc Natl Acad Sci U S A. 2018;115(31):8003–8. 30012613 10.1073/pnas.1720520115 PMC6077739

[CR15] Gimza BD, Larias MI, Budny BG, Shaw LN. Mapping the global network of extracellular protease regulation in Staphylococcus aureus. mSphere. 2019;4(5):e00676. 31645429 10.1128/mSphere.00676-19 PMC6811363

[CR16] Gottesman S. Proteolysis in bacterial regulatory circuits. Annu Rev Cell Dev Biol. 2003;19:565–87. 14570582 10.1146/annurev.cellbio.19.110701.153228

[CR17] Gottesman S, Roche E, Zhou Y, Sauer RT. The ClpXP and clpap proteases degrade proteins with carboxy-terminal peptide Tails added by the SsrA-tagging system. Genes Dev. 1998;12(9):1338–47. 9573050 10.1101/gad.12.9.1338 PMC316764

[CR18] Hackl MW, Lakemeyer M, Dahmen M, Glaser M, Pahl A, Lorenz-Baath K, Menzel T, Sievers S, Bottcher T, Antes I, Waldmann H, Sieber SA. Phenyl esters are potent inhibitors of caseinolytic protease P and reveal a Stereogenic switch for deoligomerization. J Am Chem Soc. 2015;137(26):8475–83. 26083639 10.1021/jacs.5b03084

[CR19] Jelsbak L, Ingmer H, Valihrach L, Cohn MT, Christiansen MH, Kallipolitis BH, Frees D. The chaperone ClpX stimulates expression of Staphylococcus aureus protein A by rot dependent and independent pathways. PLoS ONE. 2010;5(9):e12752. 20856878 10.1371/journal.pone.0012752 PMC2939077

[CR20] Jiang F, Chen Y, Yu J, Zhang F, Liu Q, He L, Musha H, Du J, Wang B, Han P, Chen X, Tang J, Li M, Shen H. Repurposed Fenoprofen targeting SaeR attenuates Staphylococcus aureus virulence in Implant-Associated infections. ACS Cent Sci. 2023;9(7):1354–73. 37521790 10.1021/acscentsci.3c00499 PMC10375895

[CR21] Kahan R, Worm DJ, de Castro GV, Ng S, Barnard A. Modulators of protein-protein interactions as antimicrobial agents. RSC Chem Biol. 2021;2(2):387–409. 34458791 10.1039/d0cb00205d PMC8341153

[CR22] Karamac M, Koleva L, Kancheva VD, Amarowicz R. The structure-antioxidant activity relationship of ferulates. Molecules. 2017;22(4):527. 28346342 10.3390/molecules22040527 PMC6154093

[CR23] Kim YI, Burton RE, Burton BM, Sauer RT, Baker TA. Dynamics of substrate denaturation and translocation by the ClpXP degradation machine. Mol Cell. 2000;5(4):639–48. 10882100 10.1016/s1097-2765(00)80243-9

[CR24] Kim SY, Koo YK, Koo JY, Ngoc TM, Kang SS, Bae K, Kim YS, Yun-Choi HS. Platelet anti-aggregation activities of compounds from Cinnamomum cassia. J Med Food. 2010;13(5):1069–74. 20828311 10.1089/jmf.2009.1365

[CR25] Kwon HY, Ogunniyi AD, Choi MH, Pyo SN, Rhee DK, Paton JC. The ClpP protease of Streptococcus pneumoniae modulates virulence gene expression and protects against fatal Pneumococcal challenge. Infect Immun. 2004;72(10):5646–53. 15385462 10.1128/IAI.72.10.5646-5653.2004 PMC517602

[CR26] Maurizi MR, Thompson MW, Singh SK, Kim SH. Endopeptidase clp: ATP-dependent Clp protease from Escherichia coli. Methods Enzymol. 1994;244:314–31. 7845217 10.1016/0076-6879(94)44025-5

[CR27] Mei JM, Nourbakhsh F, Ford CW, Holden DW. Identification of Staphylococcus aureus virulence genes in a murine model of bacteraemia using signature-tagged mutagenesis. Mol Microbiol. 1997;26(2):399–407. 9383163 10.1046/j.1365-2958.1997.5911966.x

[CR28] Michalik S, Bernhardt J, Otto A, Moche M, Becher D, Meyer H, Lalk M, Schurmann C, Schluter R, Kock H, Gerth U, Hecker M. Life and death of proteins: a case study of glucose-starved Staphylococcus aureus. Mol Cell Proteom. 2012;11(9):558–70. 10.1074/mcp.M112.017004 PMC3434780 22556279

[CR29] Michel A, Agerer F, Hauck CR, Herrmann M, Ullrich J, Hacker J, Ohlsen K. Global regulatory impact of ClpP protease of Staphylococcus aureus on Regulons involved in virulence, oxidative stress response, autolysis, and DNA repair. J Bacteriol. 2006;188(16):5783–96. 16885446 10.1128/JB.00074-06 PMC1540084

[CR30] Moreira W, Ngan GJ, Low JL, Poulsen A, Chia BC, Ang MJ, Yap A, Fulwood J, Lakshmanan U, Lim J, Khoo AY, Flotow H, Hill J, Raju RM, Rubin EJ, Dick T. Target mechanism-based whole-cell screening identifies bortezomib as an inhibitor of caseinolytic protease in mycobacteria. mBio. 2015;6(3):e00253–15. 25944857 10.1128/mBio.00253-15 PMC4436076

[CR31] Panossian A, Mamikonyan G, Torosyan M, Gabrielyan E, Mkhitaryan S. Analysis of aromatic aldehydes in brandy and wine by high-performance capillary electrophoresis. Anal Chem. 2001;73(17):4379–83. 11569834 10.1021/ac0014818

[CR32] Rathore S, Sinha D, Asad M, Bottcher T, Afrin F, Chauhan VS, Gupta D, Sieber SA, Mohmmed A. A cyanobacterial Serine protease of plasmodium falciparum is targeted to the apicoplast and plays an important role in its growth and development. Mol Microbiol. 2010;77(4):873–90. 20545854 10.1111/j.1365-2958.2010.07251.x

[CR33] Shoji M, Cui L, Iizuka R, Komoto A, Neoh HM, Watanabe Y, Hishinuma T, Hiramatsu K. WalK and ClpP mutations confer reduced Vancomycin susceptibility in Staphylococcus aureus. Antimicrob Agents Chemother. 2011;55(8):3870–81. 21628539 10.1128/AAC.01563-10 PMC3147622

[CR34] Tong SY, Davis JS, Eichenberger E, Holland TL, Fowler VG. Jr. Staphylococcus aureus infections: epidemiology, pathophysiology, clinical manifestations, and management. Clin Microbiol Rev. 2015;28(3):603–61. 26016486 10.1128/CMR.00134-14 PMC4451395

[CR35] Vestergaard M, Frees D, Ingmer H. Antibiotic resistance and the MRSA problem. Microbiol Spectr. 2019;7(2):10. 10.1128/microbiolspec.gpp3-0057-2018 PMC11590431 30900543

[CR36] Xu L, Zhou Y, Niu S, Liu Z, Zou Y, Yang Y, Feng H, Liu D, Niu X, Deng X, Wang Y, Wang J. A novel inhibitor of monooxygenase reversed the activity of tetracyclines against tet(X3)/tet(X4)-positive bacteria. EBioMedicine. 2022;78:103943. 35306337 10.1016/j.ebiom.2022.103943 PMC8933826

[CR37] Yi B, Hu L, Mei W, Zhou K, Wang H, Luo Y, Wei X, Dai H. Antioxidant phenolic compounds of cassava (Manihot esculenta) from Hainan. Molecules. 2011;16(12):10157–67. 22157579 10.3390/molecules161210157 PMC6264345

[CR38] Zhang J, Liu H, Zhu K, Gong S, Dramsi S, Wang YT, Li J, Chen F, Zhang R, Zhou L, Lan L, Jiang H, Schneewind O, Luo C, Yang CG. Antiinfective therapy with a small molecule inhibitor of Staphylococcus aureus sortase. Proc Natl Acad Sci U S A. 2014;111(37):13517–22. 25197057 10.1073/pnas.1408601111 PMC4169930

[CR39] Zhang Y, Zhao L, Guo X, Li C, Li H, Lou H, Ren D. Chemical constituents from Phyllanthus emblica and the cytoprotective effects on H2O2-induced PC12 cell injuries. Arch Pharm Res. 2016;39(9):1202–11. 24993870 10.1007/s12272-014-0433-2

[CR40] Zhao BB, Li XH, Zeng YL, Lu YJ. ClpP-deletion impairs the virulence of Legionella pneumophila and the optimal translocation of effector proteins. BMC Microbiol. 2016;16(1):174. 27484084 10.1186/s12866-016-0790-8 PMC4969725

